# Grynfeltt Hernia: A Deceptive Lumbar Mass with a Lipoma-Like Presentation

**DOI:** 10.1155/2015/954804

**Published:** 2015-11-30

**Authors:** Jonathan R. Zadeh, Jessica L. Buicko, Chetan Patel, Robert Kozol, Miguel A. Lopez-Viego

**Affiliations:** ^1^Charles E. Schmidt College of Medicine, Florida Atlantic University, 777 Glades Road, Boca Raton, FL 33431, USA; ^2^University of Miami-JFK Medical Center, 5301 S. Congress Avenue, Atlantis, FL 33462, USA

## Abstract

The Grynfeltt-Lesshaft hernia is a rare posterior abdominal wall defect that allows for the herniation of retro- and intraperitoneal structures through the upper lumbar triangle. While this hernia may initially present as a small asymptomatic bulge, the defect typically enlarges over time and can become symptomatic with potentially serious complications. In order to avoid that outcome, it is advisable to electively repair Grynfeltt hernias in patients without significant contraindications to surgery. Due to the limited number of lumbar hernioplasties performed, there has not been a large study that definitively identifies the best repair technique. It is generally accepted that abdominal hernias such as these should be repaired by tension-free methods. Both laparoscopic and open techniques are described in modern literature with unique advantages and complications for each. We present the case of an unexpected Grynfeltt hernia diagnosed following an attempted lipoma resection. We chose to perform an open repair involving a combination of fascial approximation and dual-layer polypropylene mesh placement. The patient's recovery was uneventful and there has been no evidence of recurrence at over six months. Our goal herein is to increase awareness of upper lumbar hernias and to discuss approaches to their surgical management.

## 1. Introduction

The Grynfeltt-Lesshaft hernia is a rare abdominal wall defect that occurs due to weakening of the transversalis fascia and the aponeurosis of the transversus abdominis [1]. This defect allows abdominal contents to protrude through the superior lumbar triangle, a region defined medially by the quadratus lumborum muscle, inferiorly by the internal oblique muscle, and superiorly by the 12th rib. Contents of this upper lumbar hernia may include retroperitoneal organs such as the kidneys, and ascending or descending colon, intraperitoneal organs such as the small bowel, stomach, and spleen, and retroperitoneal or omental adipose tissue [2].

Depending on the contents of the hernia, a patient may present with an asymptomatic lumbar mass, a lumbar mass with back pain, or a lumbar mass with vague abdominal symptoms. While the defect may be of congenital or acquired etiology, the majority of these are primary acquired hernias. They typically occur in the presence of risk factors such as advanced age, obesity, muscular atrophy, and chronic obstructive pulmonary disease or any other condition that produces persistently increased intra-abdominal pressure [3]. We present a case of a Grynfeltt-Lesshaft hernia diagnosed after the attempted resection of what was thought to be a lipoma. The patient underwent a successful surgery for the hernia involving a dual-layer mesh repair that was without subsequent complications.

## 2. Case Report

A 64-year-old female presented to the office with the complaint of a back mass. When asked to identify the location of the mass, she pointed to her right scapular line just below the costal margin. The patient reported that she first noticed this mass approximately one year prior to that visit. While it had not noticeably increased in size over the last year, the mass had recently started to produce dull pain.

The patient's past medical history was notable only for gastroesophageal reflux disease, hypercholesterolemia, and an overweight BMI of 29.1. When prompted, she denied any history of back trauma or back surgery. During the patient's physical examination, it was difficult to identify the mass at the location she indicated. After palpation in various positions, the mass was finally identified when she was instructed to stand and flex her back forward. In this position, a mass approximately 5 cm in diameter was palpable several centimeters inferior to the point the patient had indicated. The mass was soft and mobile with a smooth border. It was not found to be reducible. Palpation did not produce any pain during this examination.

At the time of this office visit, it was decided that the diagnosis of lipoma was appropriate for the patient's back mass. This decision was based on her physical findings as well as an understanding of the relatively high incidence of lipoma for individuals with this patient's chief complaint. Excision of the suspected lipoma was felt to be indicated as the mass was producing discomfort and was believed to be located such that it could be accessed by a minimally invasive approach. During preoperative examination on the day of the surgery, the patient's lumbar mass was once again challenging to identify. Palpation and marking of the mass prior to the procedure could eventually be achieved by placing the patient on her left side with her legs flexed up toward her chest. Palpation of the lumbar mass did not reveal tenderness and the exam was otherwise normal with no identified contraindications to surgery.

Following induction of anesthesia and draping of the patient, an incision was made at the marked location below the 12th rib. After dissection through the skin and subcutaneous tissue to the muscle, the mass could neither be visualized nor palpated. At this point, it was decided that it may be harmful to the patient to continue exploration without confidence in the location of the mass. Accordingly, the procedure was promptly terminated and the incision was closed and dressed appropriately.

**Figure 1:**
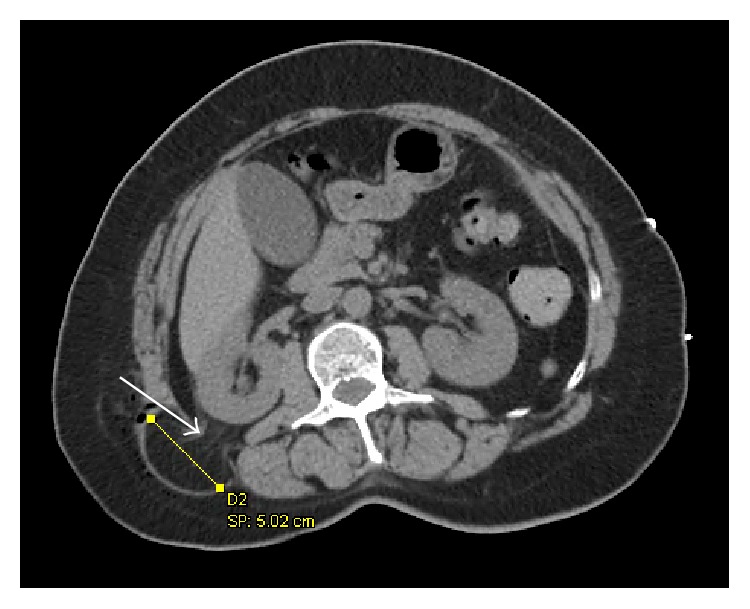
Noncontrast CT of patient's abdomen showing a right sided Grynfeltt-Lesshaft hernia. Arrow: adipose tissue herniating through defect at the upper lumbar triangle. Line with square endpoints: hernia sac diameter measurement.

The patient was then sent for a CT scan of her abdomen which identified a 5-6 cm right upper lumbar hernia sac containing retroperitoneal fat (Figure 1). The fascial defect appeared to be 1.6 cm in diameter. There was no kidney or bowel herniating into the sac. Our diagnosis after this imaging study was that of a Grynfeltt-Lesshaft lumbar hernia.

**Figure 2:**
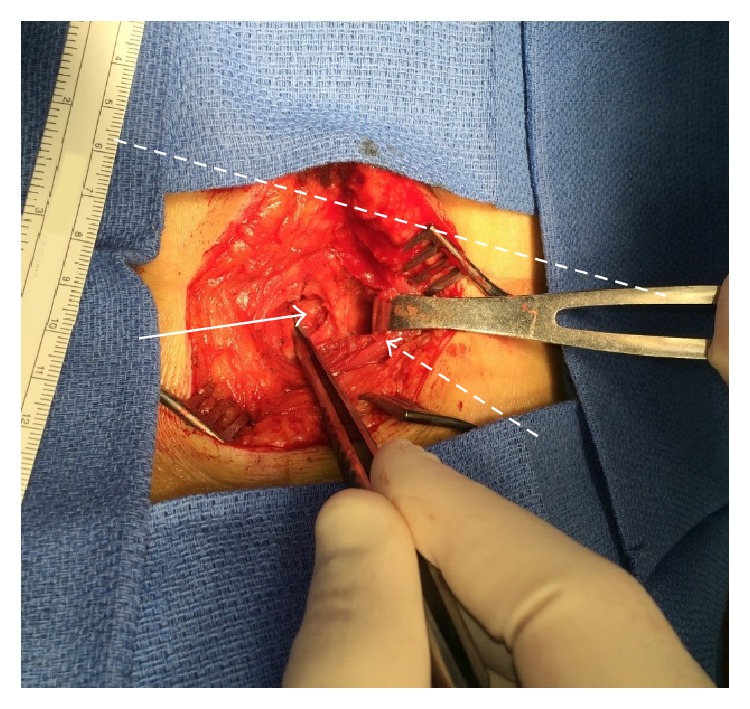
Defect following reduction of patient's hernia. Dashed line: approximate location of right 12th rib. Dashed arrow: anterolaterally retracted latissimus dorsi. Solid arrow: adipose tissue reduced through defect.

A new surgery was scheduled for the repair of the patient's hernia. At the start of the procedure, the horizontal skin incision present below the 12th rib from the attempted lipoma resection was opened and dissection was carried out to expose the latissimus dorsi. A plane was then created under this muscle by blunt dissection and the muscle was retracted anterolaterally to reveal herniated retroperitoneal fat protruding through a 1.5 cm diameter defect in the upper lumbar triangle (Figure 2). After the hernia was detached from the deep side of the transversalis fascia, a 4.3 cm diameter circular polypropylene mesh patch designed for umbilical hernia repairs was placed under the fascia. This was anchored to the deep side of the fascia with interrupted sutures circumferentially placed 2 cm from the edges of the defect. The edges were then approximated and the defect was directly sutured closed over the mesh. At this point, a 4 cm strip of polypropylene mesh was placed over the closed defect and secured to the fascia with interrupted sutures. The latissimus dorsi was then restored to its normal position and closure of the overlying tissues was performed. The patient was discharged the same day. No recurrences of the hernia or complications have been noted on six-month follow-up.

## 3. Discussion

Neither the Grynfeltt-Lesshaft hernia of the superior lumbar triangle nor the Petit hernia of the inferior lumbar triangle is the first diagnosis that comes to mind when a patient presents with a back mass. Given their rarity and their nonspecific presentation, lumbar hernias are an easy diagnosis to overlook. The physical exam for a Grynfeltt hernia may be misleading in itself as the relatively large overlaying muscle of the latissimus dorsi can make it difficult to identify the mass. Even if the bulge produced by the hernia may be palpated, the overlying muscle can interfere with the identification of specific characteristics of the hernia such as shape, firmness, and reducibility. Due to these factors, a Grynfeltt hernia may easily be confused with a more common pathology in the differential diagnosis for a back mass such as a lipoma. Our patient's findings appeared to be in line with the criteria for that diagnosis as her physical exam revealed a soft, nontender, nonreducible subcutaneous mass with a history of slow growth [4]. Furthermore, ultrasonography of the affected area showed a well circumscribed mass consistent with adipose tissue. This radiological finding is also generally suggestive of the diagnosis of lipoma [5]. All of this contributed to our patient's initial diagnosis. For physicians facing a similar diagnostic scenario, a CT study may be an appropriate option to confirm or rule out the suspected etiology of the back mass and could help to avoid delayed lumbar hernia identification.

Lumbar hernias may only account for approximately two percent of all abdominal hernias [6], but these pathologies are associated with an ever-present risk for incarceration. Subsequent strangulation of their contents is a documented complication and bowel involvement may lead to obstruction and necrosis [7]. Whether strangulation ensues or not, these defects will steadily increase and size and have a high probability of becoming otherwise symptomatic. Thus, it is important to diagnose Grynfeltt hernias early in their development and it is advisable to electively repair them when they are identified in patients healthy enough to undergo surgery [2, 8].

Due to the low incidence of lumbar hernias, there has not been a large study that has definitively identified the best repair technique for these defects. However, several different approaches have been suggested in surgical literature. Older techniques involved an open repair achieved by directly approximating the edges of the defect and providing reinforcement with flaps of adjacent fascial structures [2]. It is now generally accepted that most abdominal hernia repairs, including those of the lumbar triangles, should be achieved by tension-free methods [9]. Therefore, modern open approaches involve the placement of a mesh prosthesis. In these procedures, following exploration and reduction of hernia contents, the mesh patch is anchored to the fascia in the preperitoneal space such that it sufficiently overlaps the edges of the defect [10]. For the treatment of our patient, a synthetic mesh plug was the prosthetic choice as materials like this have been utilized in the past with favorable results [11]. Given the straining forces experienced by the upper lumbar triangle, it was decided that the closure would be reinforced with a second mesh overlaying the defect. This was felt to be safe as the patient lacked many of the risk factors for mesh infection such as smoking history, advanced age, and long anticipated procedure time [12].

A laparoscopic procedure was not attempted in this case, but such techniques are also described and are considered by some to be the appropriate first line treatments. Laparoscopic repairs of lumbar hernias have been shown to have some advantages with respect to the duration of hospitalization and postoperative complications, but they are associated with a greater probability of intraoperative complications [13].

The approach taken in our repair of this patient's Grynfeltt-Lesshaft hernia did not result in any technical difficulties. Necessary landmarks could be easily identified via the posterior incision and navigation around high-risk anatomy was not required. Since the procedure, the patient has not suffered from any noticeable complications and there has been no recurrence of the hernia. Based upon these observations, we presently feel that this modified mesh repair could be considered a safe and appropriate treatment for Grynfeltt-Lesshaft lumbar hernias.
